# The comparison of the effect of phenylephrine and epinephrine on penile spongy tissue in rats after experimental hypospadias surgery: a quantitative stereological study

**DOI:** 10.1186/s12894-019-0483-3

**Published:** 2019-06-20

**Authors:** Mehdi Shirazi, Mohammad-Bagher Rajabalian, Ali Noorafshan, Saied Karbalay-Doust, Zahra Jahanabadi, Faisal Ahmed

**Affiliations:** 1 Department of Urology, Shiraz University of Medical Sciences, Shiraz, Iran; 2 Histomorphometry and Stereology Research Center, Shiraz University of Medical Sciences, Zand Ave., Shiraz, 71348-45794 Iran; 3 Anatomy Department, School of Medicine, Shiraz University of Medical Sciences, Shiraz, Iran

**Keywords:** Epinephrine, Hypospadias repair, Phenylephrine, Stereology, Rat

## Abstract

**Background:**

Many factors including vasoconstrictor agents can interfere with wound healing process. This study aimed to compare the histopathological outcome of injection of two sympathomimetic drugs used during urologic surgery, including phenylephrine and epinephrine, on the structure of spongy tissue and urethra in a rat model of experimental hypospadias repair using stereological methods.

**Methods:**

Male rats were allocated into three groups. The first group underwent surgery without using any agents. The second and third groups underwent surgery with diluted phenylephrine (1:5000) and diluted epinephrine (1:100000) injection in the urethral plate before operation, respectively. Quantitative histological evaluation of all penises was performed after 3 weeks.

**Results:**

The results indicated no significant differences among the three groups regarding the vessels and urethral lumen and epithelium. However, the volumes of the spongy tissue and collagen bundles and the number of fibroblasts were significantly higher (35–55%) in surgery + phenylephrine and surgery + epinephrine groups in comparison to the surgery group (*p* < 0.05), with no preferences.

**Conclusions:**

Hypospadias repair using phenylephrine and epinephrine injection showed no adverse effects. Furthermore, they might lead to better postoperative structural outcomes without any preferences. However, further experimental and human studies are required to draw a firm conclusion.

## Background

The reconstruction procedure in hypospadias might be associated with some complications, such as dehiscence, urethrocutaneous fistulas, proximal strictures, diverticula, meatal stenosis, and bleeding [1]. Bleeding from the site of incision is one of the most important challenges during hypospadias repair. Early postoperative bleeding or oozing from the site of surgery may require immediate exploration and treatment. In some circumstances, hematoma formation at the site of operation may lead to cosmetic problems and even surgery failure [2]. However, the best method for inducing effective homeostasis without permanent tissue injury during hypospadias surgery has not been determined yet [3]. Various techniques have been used to manage this problem, including tourniquet application, use of bipolar electrocautery, and injection of vasoconstrictor agents into the site of incision. Tourniquet application and monopolar electrocautery have some limitations that could lead to pressure injury, vascular and thermal damages, scarring of the affected penile skin, and ischemic damages [3–5]. Epinephrine is a vasoconstrictor, which is commonly used to prevent or minimize bleeding during hypospadias surgery. Phenylephrine is another vasoconstrictor with more specific actions on vessels and fewer systemic adverse effects. However, administration of phenylephrine in hypospadias surgery has received less attention [6]. It has been demonstrated that applying homeostasis techniques during hypospadias repair might lead to ischemia and tissue damage on the urethral wall [3]. There is also concern that application of vasoconstrictor agents might interfere with wound repair. Therefore, the present study aims to evaluate the effects of injection of two vasoconstrictor agents, namely phenylephrine and epinephrine, on the structure of the urethra and corpus spongiosum in a rat model of hypospadias repair using stereological methods.

## Methods

### Animals

In this trial, 15 male adult Sprague-Dawley rats with average body weight of 200–220 g were prepared from the Center of Comparative and Experimental Medicine of the University. The rats were maintained in cages with a 12/12-h light-dark cycle, room temperature of 22–24 °C, humidity of 50%, and access to water and food. All animals were kept according to the Animal Care and Ethics Committee of the University (agreement license No. 1396-01-01-14,119).

### Experimental design

The animals were randomly divided into three groups (*n* = 5 per group). According to standard stereological articles, five animals were sufficient in each group [7]. The first group underwent surgery without any hemostasis techniques. The second and third groups underwent surgery with preoperative phenylephrine and epinephrine injection into the incision site, respectively.

### Vasoconstrictors

Diluted epinephrine (1:100000) and diluted phenylephrine (1:5000) were applied in this study as vasoconstrictor agents. Equal volumes (less than 0.1 ml) of the designated diluted vasoconstrictors were injected in the incision sites in the experimental groups before proceeding to surgery [6].

### Urethral incision and repair

All rats were anesthetized using intramuscular injection of ketamine (10–15 mg/kg) and xylazine (6–10 mg/kg). The site of operation was well shaved and disinfected by povidone-iodine topical solution. Then, it was draped with sterile sheets. External genitalia were exposed and a urethral catheter (24 g-yellow angiocath) was inserted into the urethra. Prior to the operation, diluted phenylephrine and epinephrine were injected under to the urothelium beside the incision line in groups 2 and 3, respectively [3]. Then, under sterile conditions, a longitudinal incision was made on the ventral surface of the penis over the mid raphe line from base to glans to open the corpus spongiosum and urethra. Afterwards, urethral repair was done with running suture (Vicryl 7–0). At the end of the procedure, the catheter was removed and oxytetracycline spray was used in place to prevent wound infection [8].

### Tissue preparation

Three weeks after the surgery, the rats after deep anesthesia by an intraperitoneal injection ketamine (100 mg/kg of body weight) and xylazine (8 mg/kg of body weight) were euthanized and the body of the penis, including two corpora cavernous and the corpus spongiosum that envelops the urethra, was cut from the basal part and was deposited in buffered formaldehyde. The length and weight of the penis were calculated and its volume “V (penis)” was measured by the immersion technique [8]. Then, according to the length of the penis, eight to twelve transverse slabs with equal distances were sectioned. After tissue processing, all slabs were embedded in paraffin. At this stage, 4 and 25 μm sections were obtained by a microtome. Then, the sections were stained with hematoxylin, eosin, and Heidenhain’s azan trichrome.

### Estimation of the volume of penile corpus spongiosum

The volume of corpus spongiosum was estimated under stereo-microscopy from the basal part to the glans of the penis. It should be noted that all penis sections were studied at the magnification of 64×. The whole volume of the corpus spongiosum was calculated using “Cavalieri’s method” [9]. Accordingly, the stereological probe (point grid) was superimposed over the image of penis sections viewed on the monitor. Then, the areas of corpus spongiosum sections “∑A” were multiplied by the penis sections thickness (T). After all, the area was estimated by the stereology software and the volume was estimated by the following formula:

$$ \mathrm{V}\ \left(\mathrm{corpus}\ \mathrm{spongiosum}\right)=\sum \mathrm{A}\ \left(\mathrm{corpus}\ \mathrm{spongiosum}\right)\times \mathrm{T} $$

### Estimating the volume density

The volume density of the penis sections was analyzed using a video microscopy system. Then, the stereological probe (point grid) was superimposed on the live microscopic image on a monitor by the software designed at the Histomorphometry and Stereology Research Center of the University. The volume density “*V*v (structure/corpus spongiosum)” of the spongy tissue, urethra (lumens and epithelium), vessels, and collagen bundles was estimated by the “point-counting technique” [8, 10, 11]. The following formula was employed:

$$ V\mathrm{v}\ \left(\mathrm{structure}/\mathrm{corpus}\ \mathrm{spongiosum}\right)=\mathrm{P}\ \left(\mathrm{structure}\right)/\mathrm{P}\ \left(\mathrm{corpus}\ \mathrm{spongiosum}\right) $$

Where ‘P(structure)’ was the number of test points falling on the structures (spongy tissue, urethral lumens, urethral epithelium, vessels, and collagen bundles) and ‘P (corpus spongiosum)’ was the total number of points put on the reference space (corpus spongiosum). The total volume of each structure was also estimated by the following formula:

$$ \mathrm{V}\ \left(\mathrm{structure}\right)=V\mathrm{v}\ \left(\mathrm{structure}/\mathrm{corpus}\ \mathrm{spongiosum}\right)\times \mathrm{V}\ \left(\mathrm{corpus}\ \mathrm{spongiosum}\right) $$

### Estimating the number of corpus spongiosum fibroblasts

The number and numerical density of corpus spongiosum fibroblasts were calculated using thick sections (25 μm) via the optical disector technique [12, 13]. The optical disector including a microscope (Numerical Aperture (*NA*) of 1.40, 100×) and a microcator with digital readout (MT12, Heidnehain, Traunreut, Germany) was set for estimating motion in the Z-direction. Every fibroblast nucleus that came into focus within the “disector height” was chosen if it was placed completely or slightly in the counting frame and did not contact the forbidden line. The fibroblasts’ density was estimated using the following formula:

$$ N\mathrm{V}\ \left(\mathrm{fibroblast}/\mathrm{corpus}\ \mathrm{spongiosum}\right)=\sum \mathrm{Q}/\left[\sum \mathrm{P}\times a\ (f)\times \mathrm{h}\right] $$

Where ∑Q was the number of fibroblasts computed inside the sampling frame, ∑P was number of disectors, *a* (*f*) = 774 μm^2^ was the region of the unbiased counting frame, and h = 15 μ m was the altitude of the disector. Indeed, the upper and lower 4 μm of each section were considered as guard zones by a microcator. The total number of the fibroblasts was estimated by multiplying the numerical density by V (corpus spongiosum). The fibroblasts were studied under the magnification of 1560× using a 40× oil immersion lens.

### Statistical analysis

The data were analyzed using one-way Analysis of Variance (ANOVA) followed by Tukey’s post-hoc test. *P*-values < 0.05 were considered to be statistically significant. All statistical analyses were performed using the SPSS statistical software, version 20.0 (IBM, Armonk, NY, USA program).

## Results

### Quantitative changes

**Fig. 1:**
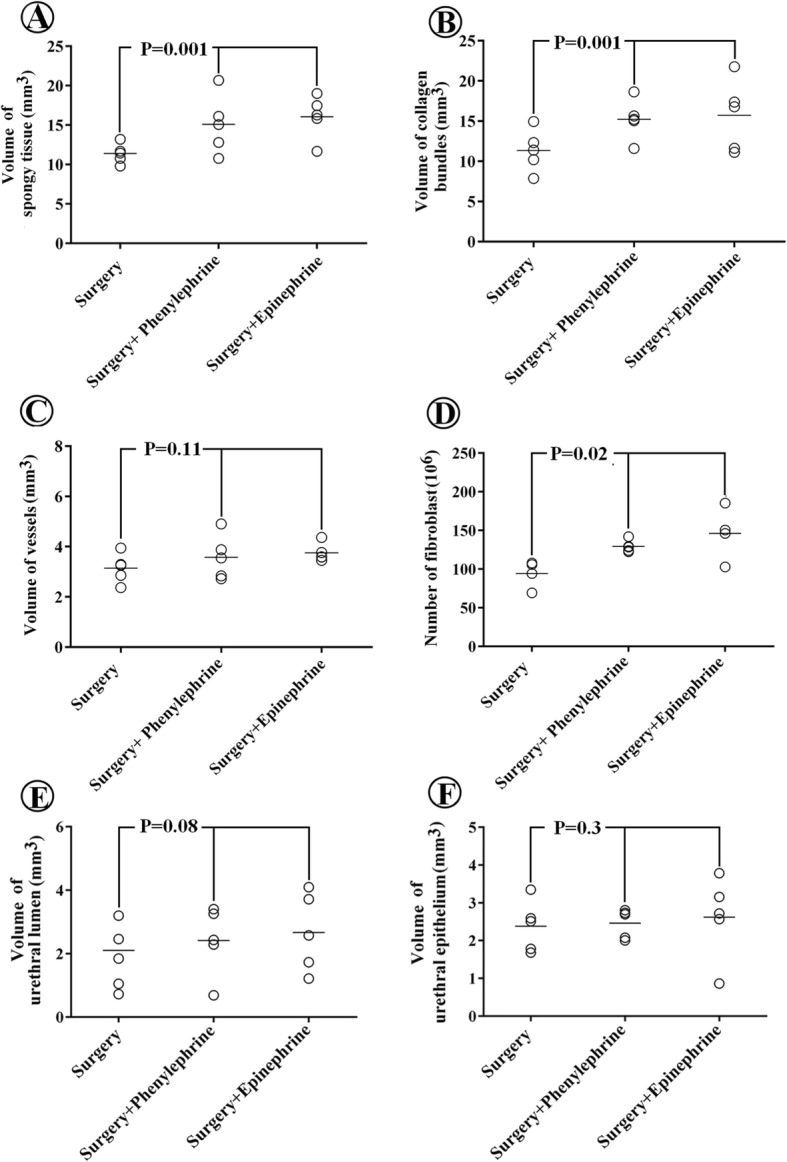
The aligned dot plot of the volumes of the spongy tissue (**a**), collagen bundles (**b**), and vessels (**c**), number of fibroblasts (**d**), and volumes of urethral lumen (**e**) and urethral epithelium (**f**) in the surgery, surgery + phenylephrine, and surgery + epinephrine groups. Each dot represents an animal and the horizontal bar is the average of the mentioned parameters in the experimental groups (*p* < 0.05 or *p* > 0.05)

The total volume of the spongy tissue and collagen bundles increased by respectively 35 and 42% in the surgery + phenylephrine group and 35 and 40% in the surgery + epinephrine group in comparison to the surgery group (*p* < 0.05) (Fig. 1).

The total number of the fibroblasts increased by 37% in the surgery + phenylephrine group and by 55% in the surgery + epinephrine group in comparison to the surgery group (p < 0.05) (Fig.1).

The data showed that the volumes of the microvessels and urethral lumen and epithelium remained constant in the surgery, surgery + phenylephrine, and surgery + epinephrine groups (*p* > 0.05) (Fig.1). However, no significant differences were found between the surgery + phenylephrine and surgery + epinephrine groups regarding the volumes of spongy tissue, vessels, urethral lumen and epithelium, and collagen bundles and the number of fibroblasts (p > 0.05) (Fig.1).

### Qualitative changes

**Fig. 2:**
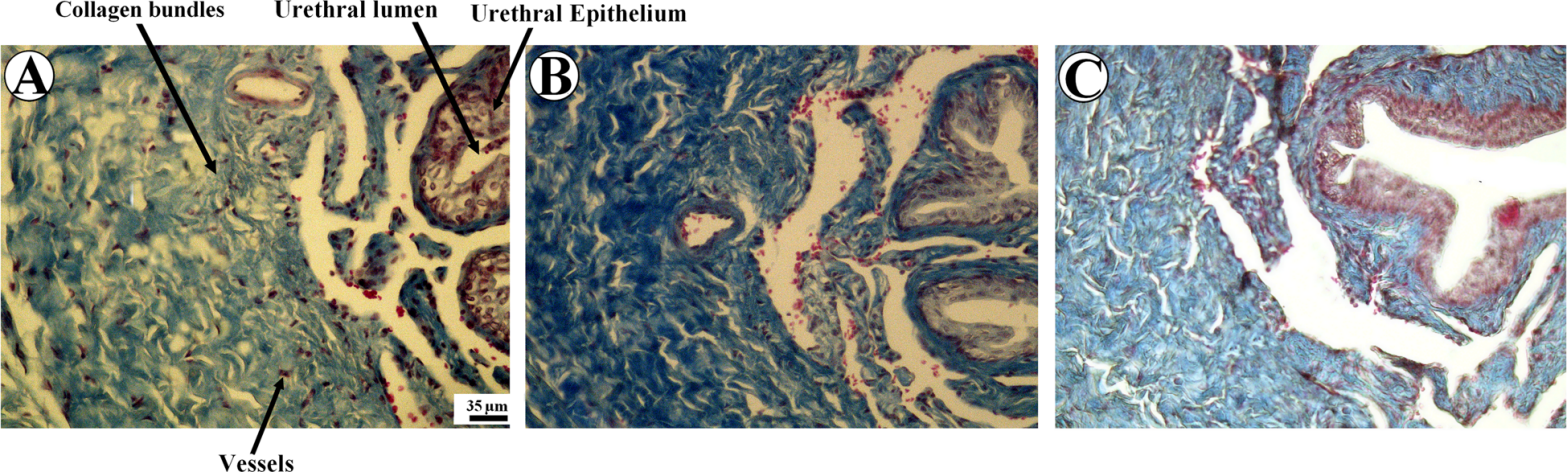
The micrographs of the penis sections in the experimental hypospadias (surgery, (**a)**), surgery + phenylephrine (**b**), and surgery + epinephrine (**c**) groups. Greater volumes of collagen bundles were observable in the surgery + phenylephrine and surgery + epinephrine groups. The microvessels and urethral lumen and epithelium have been shown on the figure. Scale bars = 35 μm. Heidenhain’s azan trichrome stain

Qualitative evaluation of the spongy tissue and the urethra has been presented in Fig. 2. After experimental hypospadias surgery, higher volumes of collagen bundles and larger populations of fibroblasts were seen in the histological sections of the surgery + phenylephrine and surgery + epinephrine groups compared to the surgery group.

## Discussion

The surgical procedure performed to treat hypospadias must provide the basic expectations, such as a vertical meatus in the middle aspect of the glans, directed urinary stream, and a straightened penis at the time of erection. Yet, maintaining the healthy structure of the penis should be the main goal [14, 15].

Providing a bloodless operative field for the surgeon is a great challenge during hypospadias repair. If this goal is achieved, it will result in a better cosmetic outcome and decrease the failure of surgery. Various techniques, such as the use of tourniquet, cauterization, and injection of vasoconstrictor agents into the site of incision, are available to manage this problem [16–18]. Kajbafzadeh et al. evaluated the effects of different hemostasis techniques on the urethral wall structure in a rabbit model of hypospadias repair. Their assessment on ultra-structural injuries, apoptotic damages, and tissue fibrosis indicated that ultra-structural and cellular damage in the urethral wall were more prominent following epinephrine hemostasis [3]. Cakmak et al. also compared tourniquet application and epinephrine injection to penile skin and concluded that epinephrine injection to penile skin might exert a deleterious effect on wound healing [19]. However, the present study findings showed that epinephrine usage in hypospadias surgery improved some stereological parameters, including a greater number of fibroblasts and collagen bundles in the spongy tissue postoperatively. Another study by Alizadeh et al. also revealed that epinephrine injection decreased bleeding without any significant harmful effects on postoperative clinical outcomes in hypospadias surgery [17].

The current study demonstrated the advantages of using injected vasoconstrictor agents to obtain a better stereological outcome compared to the surgery group following hypospadias surgery in rats. Vasoconstrictor drugs constrict blood vessels and decrease perfusion to the site of injection. Thus, bleeding decreases at their administration site [6]. In the present study, epinephrine and phenylephrine were applied as vasoconstrictors. Epinephrine is a vasoconstrictor used commonly for preventing or minimizing bleeding during surgical procedures. Epinephrine has agonist effects on both alpha and beta adrenergic receptors, but it exerts its vasoconstrictor action through affecting alpha adrenergic receptors. When epinephrine tissue levels decrease over time, the primary effect on the vessels will revert to vasodilation, as beta effects predominate. This may lead to increased postoperative blood loss. On the other hand, phenylephrine is a vasoconstrictor with almost pure alpha agonist effects, resulting in few systemic side effects. Indeed, phenylephrine has a long duration of action compared to epinephrine. Therefore, the postoperative period passes with less bleeding and total blood loss is usually lower when phenylephrine is used [6]. Unfortunately, less attention has been paid to the use of phenylephrine in hypospadias surgery. However, it is used in priapism treatment in which vasoconstrictor effect is required. Burnett and Bivalaqua reported that phenylephrine was the preferred vasoconstrictor for treatment of priapism because of its lower risk profile for systemic cardiovascular adverse effects [20]. Moreover, Montague et al. recommended that if phenylephrine was unavailable for treatment of priapism, other alpha adrenergic agonists, such as epinephrine, norepinephrine, and ephedrine, could be used [21].

The present study compared the histopathological outcomes of injecting phenylephrine and epinephrine on the spongy tissue and the urethra. According to the results, phenylephrine and epinephrine injection led to a better stereological outcome compared to the surgery group following hypospadias surgery in rats. Similarly, Najeh et al. demonstrated that epinephrine solution 1:100000 was safe and decreased bleeding and wound hematoma in hypospadias surgery. It also eliminated the need for tourniquet, reduced the use of bipolar cautery, and decreased the duration of operation and admission [2].

The current study had some limitations. Firstly, the animals undergoing surgery did not have hypospadias. Therefore, actual hypospadias models were not available and normal urethras were incised. Secondly, clinical outcomes, such as total bleeding during surgery, wound hematoma, and urethrocutaneous fistula formation, were not assessed after the surgery. Thirdly, adverse effects, including tachycardia and hypertension, were not evaluated. Finally, the utilized method was not compared to other hemostasis techniques, such as tourniquet application. Thus, further experimental and human studies are needed to draw firm conclusions. Future evaluations may also be required to compare phenylephrine and epinephrine in terms of systemic adverse effects and clinical outcomes in hypospadias surgery in order to determine which one can be the best option for achieving hemostasis during this surgery.

## Conclusions

The study findings revealed that hypospadias repair using the aforementioned vasoconstrictors resulted in better postoperative stereological outcomes compared to surgery without using any hemostasis techniques. Besides, no stereological differences were observed between phenylephrine and epinephrine.

## Data Availability

The datasets analyzed during the current study are available from the corresponding author on reasonable request.
